# Clinicopathological Spectrum of Syringoma: A Report of 50 Cases From a Tertiary Care Hospital in Eastern India

**DOI:** 10.7759/cureus.32694

**Published:** 2022-12-19

**Authors:** Divya Tiwary, Mala Mukherjee, Asitava Deb Roy, Himel Mondal

**Affiliations:** 1 Pathology, Mata Gujri Memorial Medical College, Kishanganj, IND; 2 Pathology, All India Institute of Medical Sciences, Deoghar, Deoghar, IND; 3 Physiology, All India Institute of Medical Sciences, Deoghar, Deoghar, IND

**Keywords:** eccrine duct, clinical pathology, bihar, benign adnexal tumours, histopathology examination, fine needle aspiration cytology (fnac), skin pigmentation, epithelial cells, sweat, syringoma

## Abstract

Background

Syringoma is a benign adnexal neoplasm and is considered safe with very low malignant potential. However, multiple tiny lesions typically affect the face and exposed area, which may cause a cosmetic concern for the patient. After a clinical diagnosis, there are two methods to diagnose syringoma: fine needle aspiration cytology (FNAC) and histopathology. FNAC is generally used for the initial evaluation of syringoma, while histopathology is used as a confirmatory test to diagnose syringoma. In developing and resource-limited settings, the combination of FNAC and histopathology would cause a financial and logistics burden.

Objective

This study aimed to observe the cytological and histopathological features of cases clinically diagnosed as syringomas in a tertiary care hospital to suggest the use of either FNAC or histopathology for diagnosing syringoma.

Materials and Methods

This cross-sectional observational study was conducted in the Department of Dermatology and Department of Pathology of a tertiary care hospital in eastern India from November 2021 to April 2022. Any clinically provisionally diagnosed case of syringoma was recruited for the study after obtaining informed consent for voluntary participation. With aseptic precautions, the tissue aspirates and punch biopsy were obtained in the Department of Dermatology and the samples were sent to the Department of Pathology. Cytological and histological examination was conducted by a single expert pathologist.

Result

A total of 50 cases (36 female, 14 male) with a median age of 23 years (range 10-40 years) were included in the study. A total of 43 cases were presented with papular lesions and seven with nodules. In the majority of the cases (40%), the lesion was in the eyelid followed by 26% in the arm. In FNAC, 22 cases were found to be benign adnexal lesions, 16 were suggestive of syringoma, eight were diagnosed as xanthoma, two were diagnosed as warts, and two cases were inadequate for opinion. Histologically, 42 cases were confirmed as syringoma, six were diagnosed as xanthoma, and two cases were diagnosed as warts. There was a significant difference between diagnosis by FNAC and histopathology (McNemar χ^2^ = 24.038, p-value = 0.0001).

Conclusion

We found that FNAC and histopathological diagnosis of syringoma may not be corroborative. Benign adnexal lesions are difficult to categorize by FNAC. Histopathological examination of clinically diagnosed cases of syringoma is of help for definitive diagnosis. Hence, FNAC may be avoided for saving time and discomfort for the patients and clinically diagnosed cases may be diagnosed by histopathological examination.

## Introduction

Syringoma is a benign adnexal tumor that develops from the intraepidermal part of the eccrine sweat ducts. It is more common in females during puberty [1]. Patients suffering from syringoma present with tiny, firm, and skin-colored papules in their eyelids, face, axilla, chest, breast, and other areas of the body. The lesions are commonly symmetrically distributed in body parts and are often asymptomatic. Although a single lesion measures about 1-3 mm in diameter, a large number of such lesions may be a cosmetic concern [2]. There are four clinical types of syringoma: (i) localized, (ii) familial, (iii) linked to Down's syndrome, and (iv) generalized, which includes numerous and eruptive syringomas [3].

Fine needle aspiration cytology (FNAC) is a less invasive procedure where only aspirate is collected from the lesion. Due to convenience in sample collection in an outpatient department setting, many dermatologists suggest FNAC for the initial diagnosis of most nodular lesions. However, in general, the FNAC finding may not be conclusive for a diagnosis. In such cases, a histopathological examination of the tissue of the lesion becomes necessary. In syringoma, although the majority of the case reports show that initial evaluation was done by FNAC, the final diagnosis is made after histopathological observation [4-6].

With this background, we conducted this study in a tertiary care hospital situated in the eastern part of India to observe the pattern of FNAC and histopathological findings in clinically diagnosed cases of syringoma. The study result would help to find if the cytological and histopathological features of syringoma are corroborated.

## Materials and methods

Type and setting

This cross-sectional observational study was conducted in the Department of Pathology in collaboration with the Department of Dermatology in a tertiary care hospital, Mata Gujri Memorial Medical College, Kishanganj, Bihar, India. This study was approved by the Institutional Ethics Committee of Mata Gujri Memorial Medical College, Kishanganj, Bihar, India (approval number: IEC/09/2020). The study was conducted from November 2021 to April 2022.

Sample

We recruited patients from the Outpatient Department of Dermatology from the attached hospital. We used a convenience sample with pre-defined inclusion and exclusion criteria for this study. Any patient (all ages and gender) attending the Department of Dermatology and clinically diagnosed with syringoma for the first time was included in this study. Patients with any concurrent dermatological or other diseases were excluded from the study. Any patient receiving any modality of treatment for syringoma was also excluded from the study. Patients of all ages or genders were recruited. For adult patients (age ≥ 18 years), informed consent was obtained in written form, and where the age of the patient was < 18 years, the written consent was taken from any of the parents of the participant, and verbal assent was obtained from the research participants.

Tests

With aseptic precautions, the sample was collected from the lesions in the Department of Dermatology. For FNAC, the aspirate was collected from the lesion. Then a punch biopsy was taken. The specimens of punch biopsy were sent to the Department of Pathology in 10% neutral buffered formalin for histopathological examination. The cytological examination was conducted immediately with the smears made with tissue aspirates after staining with Leishman-Giemsa stain. The biopsy tissue was processed following standard protocols and sections were stained with hematoxylin and eosin. One experienced pathologist examined all the slides to avoid any interobserver variation. The details of dermatological abnormality were not sent along with the specimen to reduce bias. For capturing the photomicrograph, we used a smartphone camera with a homemade smartphone adapter [7].

Statistical analysis

Data were expressed descriptively in number and percentage. Categorical data were compared by the Chi-square test and McNemar test. A p-value <0.05 was considered statistically significant. For all the statistical tests, we used a guideline by Mondal et al. and conducted the tests on public domain websites providing statistical test services [8].

## Results

A total of 50 cases (36 female, 14 male) with a median age of 23 years (range 10-40 years) were analyzed in this study. A total of 24 research participants were in the age group of 10-20 years, 19 were in 21-30 years, and seven were in 31-40 years (χ^2^ = 9.18, p = 0.01). Hence, the majority of the patients in this study were in the age group of 10-20 years.

**Figure 1:**
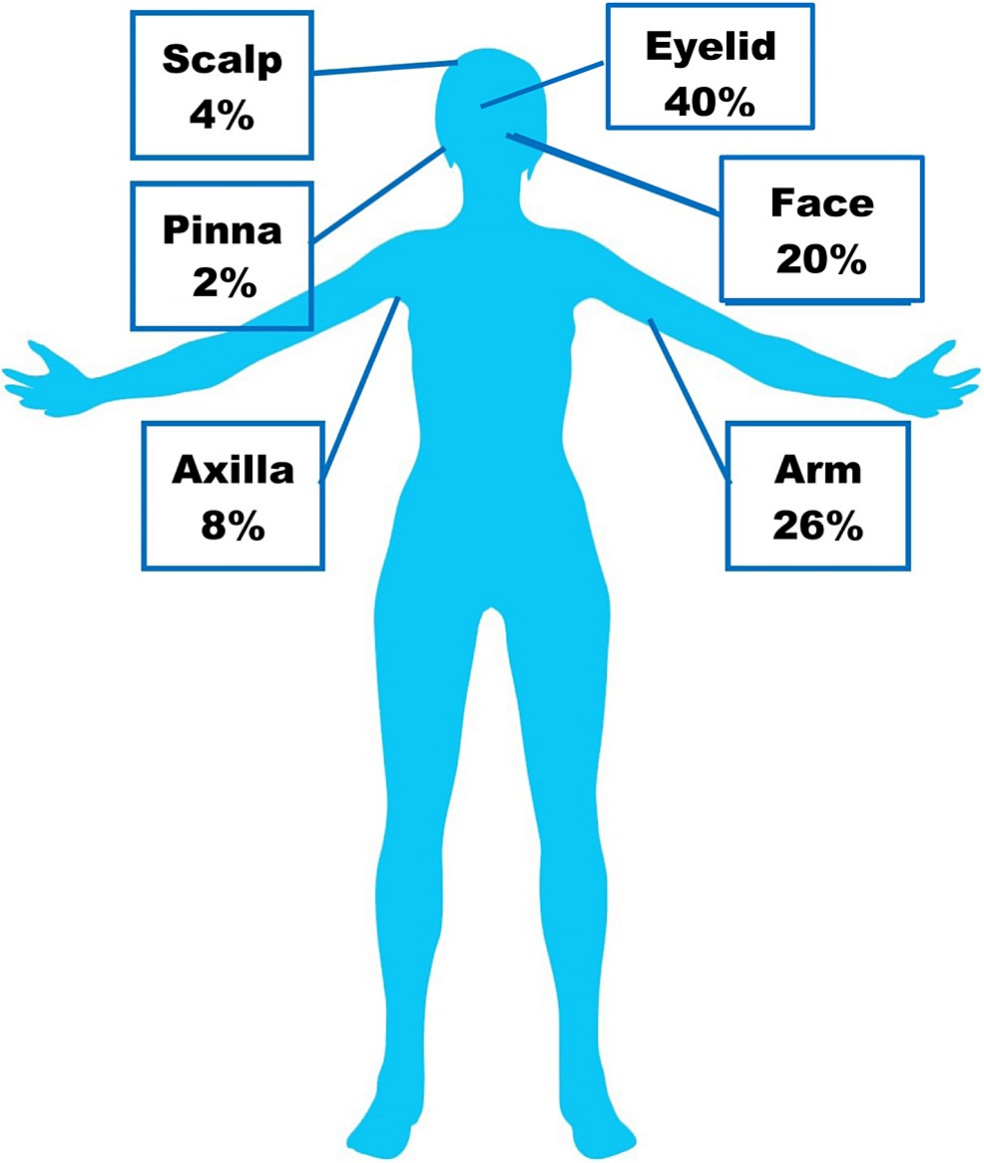
Distribution of lesions found in different parts of the body Image credit: The figure used is from Pixbay.com and has been edited by the corresponding author for exclusive use in this article.

**Figure 2:**
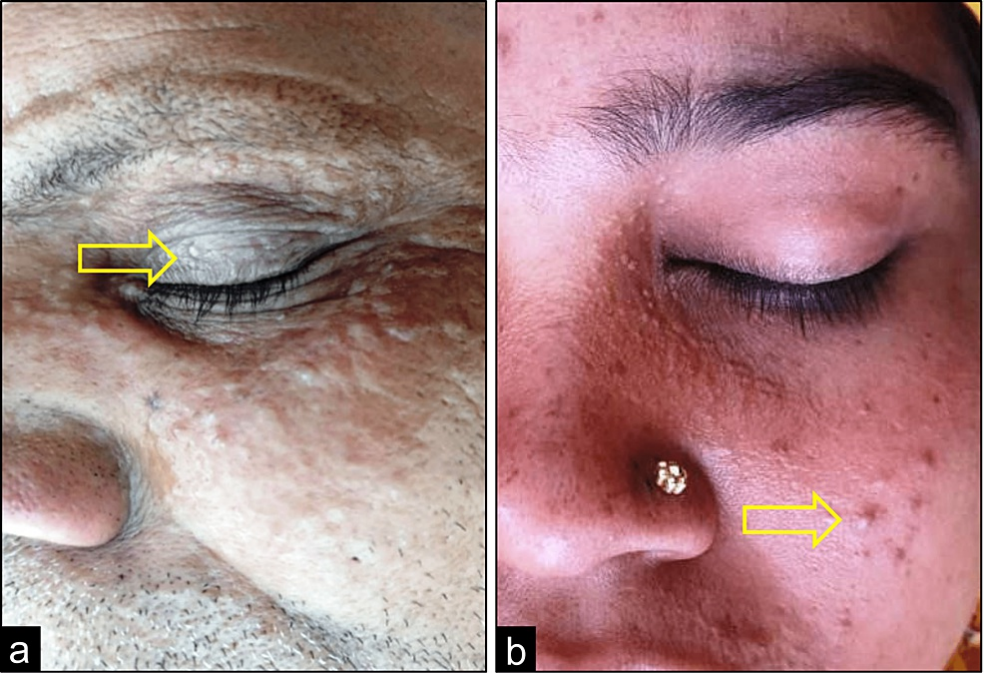
Clinically diagnosed syringoma on (a) eyelid and on (b) face

A total of 43 cases presented with papular lesions and seven with nodules. In the majority of the cases (40%), the lesion was in the eyelid followed by 26% on the arm and 20% on the face. The distribution of lesions is shown in Figure 1. Syringoma in the eyelid of a male patient is shown in Figure 2a and the lesion on the face of a female patient is shown in Figure 2b.

The FNAC findings are shown in Table 1. The majority of the cases (44%) were diagnosed as suggestive of benign adnexal lesions.

**Table 1:** Fine needle aspiration cytology finding of the cases FNAC: fine needle aspiration cytology

FNAC finding	Number (%)	χ^2^, p-value
Benign adnexal lesion	22 (44)	31.2, <0.0001
Syringoma	16 (32)	
Xanthoma	8 (16)	
Wart	2 (4)	
Inadequate for opinion	2 (4)	

The histological findings are shown in Table 2. Syringoma was diagnosed in 42 cases, xanthoma was diagnosed in six cases, and two cases were diagnosed as warts.

**Table 2:** Histopathological findings of the cases

Histological finding	Number (%)			χ^2^, p-value
Syringoma	42 (84)	Tadpole-like structure	42 (100)	58.28, <0.0001
		Fibrous stroma	35 (83.33)	
		Acanthosis	32 (76.19)	
		Basal cell pigmentation	26 (61.9)	
		Foreign body giant cells	14 (33.33)	
		Cyst	13 (30.95)	
		Clear cell change	3 (7.14)	
Xanthoma	6 (12)			
Wart	2 (4)			

**Figure 3:**
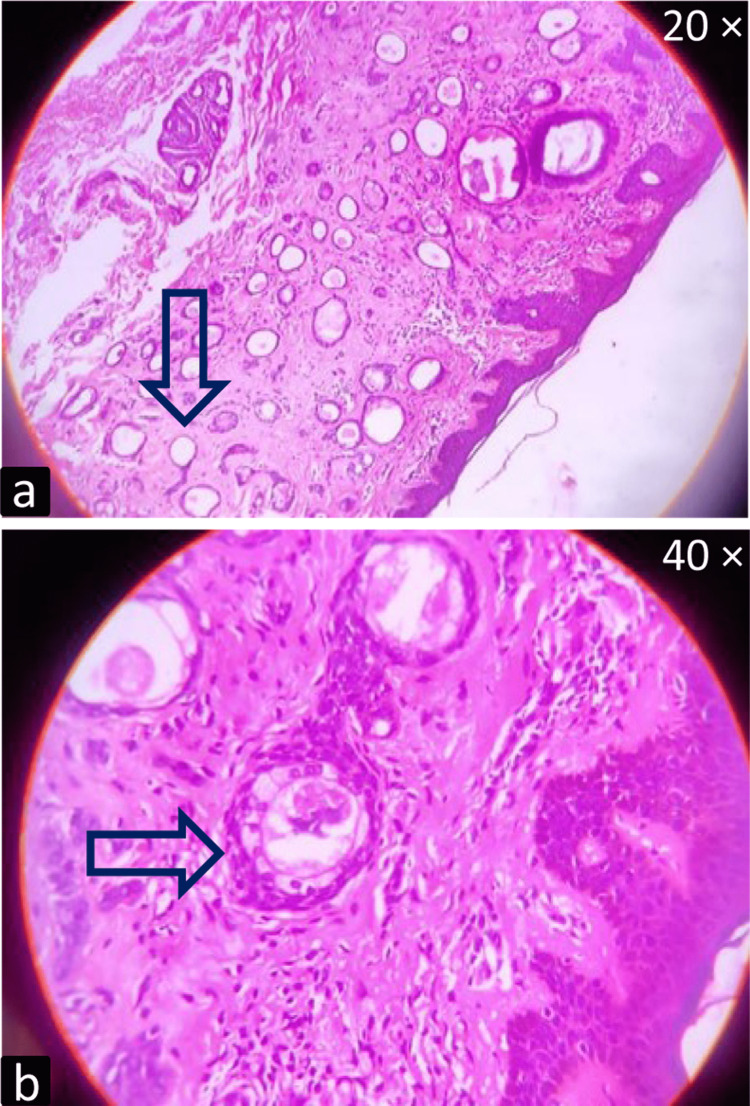
(a) Histopathological specimen (stained with hematoxylin and eosin; 20 ×) showing tadpole strands embedded in the fibrous stroma, (b) Histopathological specimen (stained with hematoxylin and eosin; 40 ×) showing eccrine gland with clear cell change Photomicrographs were taken by a smartphone camera following the free-hand technique and later cropped for presentation in this manuscript

All the cases showed tadpole-like structures under the microscope and the next common features were fibrous stroma (83.33%) and acanthosis (76.19%). Figure 3a shows a photomicrograph with a tadpole-like structure. Figure 3b shows a photomicrograph of the eccrine gland with clear cell change.

Table 3 shows the number of diagnosed cases of syringoma by FNAC and histopathological examination. There were 16 cases where the diagnosis by FNAC was corroborative with histopathological findings. However, in 26 cases, FNAC could not diagnose syringoma where histopathology confirmed the diagnosis (χ^2^ = 24.038, p = 0.0001).

**Table 3:** Diagnosed and undiagnosed number of cases in FNAC and histopathology FNAC: fine needle aspiration cytology

Test		Histopathology	
		Diagnosed	Not diagnosed
FNAC	Diagnosed	16	0
	Not diagnosed	26	8
McNemar χ^2^ (1) = 24.038, p-value = 0.0001			

## Discussion

In this study, we found that the FNAC is inconclusive for the diagnosis of syringoma cases in the majority of the cases as benign adnexal lesions are difficult to categorize by cytological examination. Twenty-two cases that were reported as suggestive of benign adnexal lesions on FNAC later turned out to be syringomas on histopathology. On histological investigation, syringoma can be definitively diagnosed as the dermis contains numerous tiny channels and epithelial cords, which may be seen by using the hematoxylin & eosin stain. The ducts are bordered by two rows of flattened epithelial cells [9]. With the support of established literature, we found nests of eccrine ducts and tadpole-like structures that were embedded in the fibrous stroma. This is corroborative to previous reports by Samia et al. and Choi et al. [10,11] The next finding was the clear cell changes of epithelial cells as shown in Figure 2b. Furthermore, a few additional findings such as acanthosis, basal cell pigmentation, and granulomatous foreign body reaction were found.

Two cases of syringoma confirmed by histopathology were inadvertently diagnosed as xanthoma on FNAC. This could have been confusion about clear cell changes in syringoma with lipid-laden macrophages in xanthoma, in the setting of a paucicellular smear. Two cases could not be reported on FNAC due to a lack of adequate material. From the punch biopsy sample, these two cases were later confirmed as syringomas on histopathology. This remains a general procedural challenge in cases of FNAC. Various reasons like lack of experience of the performer, very tiny lesions, fibrous/calcified lesions, and uncooperative patients might lead to low yield of material on aspiration.

In developing countries like India, the patient load is very high and is managed with limited resources and manpower. Hence, in government-run settings, if a patient with clinically diagnosed syringoma is sent for FNAC, it would be an unnecessary burden to the hospital resource as limited pathologists are working with a high workload and with limited materials. In addition, in the private sector, if FNAC is suggested, it would cause discomfort and extra financial burden to the patient. Hence, a dermatologist may directly think of taking a punch biopsy followed by a histopathological examination in clinically diagnosed cases of syringoma. This would help save time and resources.

Previous studies have documented the eyelid as the most common site of syringoma [3,12]. Supporting that, we also found the most common site to be the eyelid, followed by arms and face. The age distribution of patients shows that the majority of the patients were in 10-20 years of age and predominantly female. Syringoma is a benign adnexal tumor that develops from the intraepidermal eccrine duct. It mostly affects females and appears as a single papule or many papules on the eyelids during adolescence or later in life. Corroborative with other studies, we also found a higher number of females in our sample [13]. The underlying reason may be an influence of estrogen or progesterone in females. However, it is still a theoretical concept and should be explored further [12]. A higher number of cases of syringoma in females may be attributed to higher reporting by females due to cosmetic concerns [14].

An uncommon form of syringoma is clear-cell syringoma that can be diagnosed on histopathology. It develops as a result of glycogen buildup in the syringoma. Diabetes mellitus has a substantial correlation with clear-cell syringoma [15,16]. Clinically, it may not be distinguished from common syringoma. A previous study by Patrizi et al. reported only one case of clear cell variant among 29 cases of non-diabetic patients suffering from syringoma [17]. Similar to that study, we found only three cases with clear cell changes in our sample of nondiabetic patients.

Limitations

This study has several limitations. This is a single-center study with a convenience sample recruited from a tertiary care hospital. Being a non-probability sample, the inference lacks generalization. As many a time, syringoma cases are not reported frequently due to ignorance and lack of distressing symptoms, it was not possible to add more patients to this study due to logistics limitations. Further studies may be conducted in multiple centers for a more generalized result.

## Conclusions

We found that cytological and histopathological findings in clinically diagnosed cases of syringoma may not be corroborative. Hence, for confirmation of the diagnosis of syringoma, FNAC findings should be supported by histopathology. In developing countries like India, undergoing both FNAC and biopsy may cause extra financial burden to patients. Hence, our recommendation is that clinically diagnosed cases of syringoma may be examined by only histopathology for a definitive diagnosis. This would help in the diagnosis of the cases with a single test, which would save time and money, and indeed cause lesser hassles for the patient.
